# Factors associated with hypertension among patients with type 2 diabetes mellitus at Arba Minch general hospital, South Ethiopia

**DOI:** 10.1016/j.pmedr.2025.103277

**Published:** 2025-10-15

**Authors:** Biniyam Demisse Andarge, Nini Asfaw Negash, Sayih Mehari Degualem, Habtamu Esubalew Bezie, Habtamu Wondmagegn, Tsegazeab Ayele Meshesha, Maycas Dembelu Gembe, Yohannes Habtegiorgis

**Affiliations:** a College of Medicine and Health Science, Arba Minch University, Arba Minch, Ethiopia; b College of Medicine and Health Science, University of Gondar, Gondar, Ethiopia; c College of Health Science, Mettu University, Mettu, Ethiopia; d Director of Quality Services, Aleta Wondo Hospital, Aleta Wondo, Ethiopia

**Keywords:** Type 2 diabetes mellitus, hypertension, comorbidity, self-care, Ethiopia

## Abstract

**Objective:**

To assess the prevalence of hypertension and its associated factors among adults with type 2 diabetes mellitus (T2DM) at Arba Minch General Hospital, South Ethiopia.

**Methods:**

A facility-based cross-sectional study was conducted from December 1 to 30, 2024 at Arba Minch General Hospital. A total of 381 adults with T2DM were selected using systematic random sampling. Data were collected through structured interviews, medical record reviews, and physical measurements. Multivariable logistic regression was applied to identify factors associated with hypertension. Results are presented as adjusted odds ratios (AORs) with 95 % confidence intervals (CIs).

**Results:**

The prevalence of hypertension was 52.8 % (95 % CI: 47.6,57.9). Independent factors included age ≥ 40 years (AOR: 3.32; 95 % CI: 1.68,6.56), rural residence (AOR: 0.41; 95 % CI: 0.19,0.88), family history of diabetes (AOR: 2.31; 95 % CI: 1.23,4.45), diabetes duration ≥10 years (AOR: 3.50; 95 % CI: 1.87,6.65), physical inactivity (AOR: 2.53; 95 % CI: 1.01,6.55), and not using a self-monitoring device (AOR: 2.11; 95 % CI: 1.13,3.93).

**Conclusion:**

More than half of adults with T2DM had coexisting hypertension. Integrated chronic disease management focusing on lifestyle modification, regular blood pressure monitoring, and enhancing patient self-care are essential to improve outcomes in this population.

## Highlights

• Hypertension affected 52.76 % of adults with type 2 diabetes mellitus in South Ethiopia.
• Older age and longer diabetes duration were strong factors for hypertension.
• Lack of physical activity and glucometer use were also significantly associated.
• Findings support integrated chronic care and lifestyle interventions.

## 1 Introduction

Type 2 diabetes mellitus (T2DM) is a chronic metabolic disorder characterized by high blood sugar due to insulin resistance associated with progressive β-cell dysfunction (Banday et al., 2020). The global prevalence of diabetes is estimated to be 589 million adults worldwide, and it is projected to increase to 853 million by 2050. Among them, type 2 diabetes mellitus accounts for approximately 90 % of these cases (Magliano et al., 2025). In addition to hyperglycemia, patients with T2DM are also often burdened with comorbidities, including hypertension, which together increase the risk of complications (Tong et al., 2023).

Hypertension is the most common among comorbidities that can affect approximately 50–80 % of patients with T2DM (Jia and Sowers, 2021). In a study, those patients with T2DM are up to two times more likely to develop hypertension when compared to nondiabetic individuals (Naseri et al., 2022). This common coexistence increases the risk of cardiovascular diseases (CVD) such as coronary heart disease, myocardial infarction, stroke, and heart failure, and microvascular complications like nephropathy and retinopathy (Usman et al., 2021). Evidence from another study also demonstrated an increased risk of CVD and hypertension in individuals with T2DM (Wang et al., 2024). Further analysis indicated higher mortality among those who had hypertension at the time of diabetes diagnosis (Yuan et al., 2025).

The pathophysiological mechanisms linking T2DM and hypertension are complex and overlapping. Insulin resistance and high blood sugar levels increase the activity of the sympathetic nervous system, causing the kidneys to retain more sodium and water, which in turn raises blood pressure. At the same time, the blood vessels lose their ability to relax properly due to reduced nitric oxide levels, chronic inflammation, and arterial stiffening. Over time, these changes increase vascular resistance and make hypertension harder to control in diabetic patients (Jia and Sowers, 2021; Petrie et al., 2018).

Because of this interplay, the coexistence of both conditions often requires comprehensive management, including multidrug therapy, lifestyle modification, and integrated clinical management (Tong et al., 2023; De Boer et al., 2017). Multiple studies have identified factors associated with hypertension in people with T2DM. Demographic characteristics such as increasing age, sex, socioeconomic status, education, and residence, alongside behavioral and clinical factors like obesity, sedentary lifestyle, increased salt intake, poor glycemic control, dyslipidemia, and prolonged duration of diabetes, have all been shown to associate with hypertension in diabetic patients (Mariye et al., 2019; Akalu and Belsti, 2020; Taheri et al., 2024).

Double burdens of hypertension and diabetes mellitus in sub-Saharan Africa are increasing due to demographic and lifestyle changes. However, the detection and management of these chronic conditions are poor due to weak health systems, limited surveillance data, and the shortage of trained health professionals (Gafane-Matemane et al., 2024). In Ethiopia, national-level studies indicate an increasing prevalence of both conditions (Gebreyes et al., 2018). Several facility-based studies from different regions of Ethiopia also reported that a high prevalence of hypertension exists among patients with T2DM (Akalu and Belsti, 2020; Desta et al., 2024; Motuma et al., 2023; Abdissa and Kene, 2020).

However, despite the growing burden of these comorbid conditions, evidence on hypertension among patients with T2DM in Southern Ethiopia remains scarce. Differences in lifestyle, healthcare access, and socioeconomic conditions in this region may uniquely influence the burden and patterns of disease. Therefore, this study aimed to assess the prevalence and associated factors of hypertension among adults with type 2 diabetes mellitus in Southern Ethiopia. By generating evidence, the study provides a stronger rationale for local prevention and management strategies. The findings are intended to inform health professionals, hospital administrators, policymakers, and the government by highlighting the magnitude of the problem, identifying modifiable risk factors, and guiding targeted interventions. Ultimately, filling this regional evidence gap will contribute to the broader scientific understanding of diabetes–hypertension comorbidity in Ethiopia and across sub-Saharan Africa.

## 2 Materials and methods

### 2.1 Study design, period, and setting

A facility-based cross-sectional study was conducted from December 1 to 30, 2024, at Arba Minch General Hospital (AMGH) in Arba Minch Town, Gamo Zone, South Ethiopia, approximately 438 km south of Addis Ababa. AMGH is the only general hospital in the area, serving over 1 million people annually and offering a wide range of inpatient and outpatient services.

The hospital's chronic care unit provides follow-up for non-communicable diseases, including more than 1150 diabetic patients, of whom 826 are adults with a confirmed T2DM diagnosis.

### 2.2 Study population, sample size, and sampling procedures

The study population included adults with T2DM attending follow-up visits at the chronic care clinic of Arba Minch General Hospital (AMGH) during the study period. Eligible participants were aged 18 years or older, had a confirmed T2DM diagnosis, attended at least three follow-up visits, and provided informed consent. Patients with incomplete records, missing key laboratory data, Type 1 diabetes, or gestational diabetes were excluded.

The sample size was calculated using a single population proportion formula, assuming a 59.5 % prevalence of hypertension among patients with T2DM (Akalu and Belsti, 2020), a 5 % margin of error, and a 95 % confidence level. The computed sample size was:

$n=\frac{{\left(\mathrm{Za}/2\right)}^{2}\times P\left(1-P\right)}{d^{2}}$ $=\frac{{\left(1.96\right)}^{2}\times 0.595\left(1-0.595\right)}{0.05^{2}}=371$

With a 5 % non-response rate, the final sample size was 390 participants.

Systematic random sampling was used to select participants from 826 eligible patients with T2DM. A sampling interval of approximately k = 2 was calculated, and every second patient was chosen from the registration list during routine follow-up visits. Informed consent was obtained before participation. Data were collected through interviews, medical record reviews, and physical measurements.

### 2.3 Data collection procedures and measurements

We collected the data by using face-to-face interviews, medical record reviews, and physical measurements. Socio-demographic variables (age, sex, marital status, education, occupation, residence) and self-care practices (salt intake, physical activity, smoking, alcohol use, and self-monitoring) were obtained using a structured, pretested questionnaire based on validated tools (Akalu and Belsti, 2020; World Health Organization, 2017). Interviews were conducted in the local language for clarity.

Clinical and biochemical data such as fasting blood glucose, HbA1c, diabetes duration, family history, medication, and hypertension status were extracted from medical records using a checklist.

Anthropometric and blood pressure measurements were taken using standard procedures. Blood pressure was measured using a validated, automated digital sphygmomanometer (OMRON) with appropriate cuff sizes. Before measurement, participants were advised to avoid smoking, caffeine, alcohol, and vigorous physical activity for at least 30 min. They rested in a seated position with back support for at least 15 min before measurement. Three readings were taken at three-minute intervals, and the average of the second and third readings was recorded for analysis. Anthropometric measurements were performed using calibrated instruments, following WHO recommendations. Weight was measured to the nearest 0.1 kg using a digital scale, and height was measured to the nearest 0.1 cm using a stadiometer with participants standing upright, barefoot, and with heels together. BMI was then calculated as weight (kg) divided by height squared (m^2^).

Data collection was conducted by four trained nurses under the supervision of a medical doctor and the principal investigator. Medical records were reviewed to validate diagnoses and laboratory results. Daily supervision, spot checks, and random audits of completed forms were carried out to ensure data quality, accuracy, and completeness.

### 2.4 Operational definitions

#### 2.4.1 Hypertension

defined as mean systolic blood pressure (SBP) ≥140 mmHg and/or mean diastolic blood pressure (DBP) ≥90 mmHg, or individuals with a documented hypertension diagnosis who were taking antihypertensive medications (De Boer et al., 2017).

#### 2.4.2 Staging of Hypertension

The categorization is based on the JNC 7 (Joint National Committee) guidelines, defined as follows: Normal Blood Pressure: Systolic Blood Pressure (SBP) <120 mmHg and Diastolic Blood Pressure (DBP) <80 mmHg; Prehypertension: SBP 120–139 mmHg or DBP 80–89 mmHg; Stage I Hypertension: SBP 140–159 mmHg or DBP 90–99 mmHg and Stage II Hypertension: SBP ≥160 mmHg or DBP ≥100 mmHg (Chobanian et al., 2003).

#### 2.4.3 Glycemic control

Optimal glycemic control was defined as patients with an HbA1C level of <7 % and Suboptimal control was defined as an HbA1C level of 7 % or more (American Diabetes Association, 2020).

#### 2.4.4 Salt intake

Salt consumption was assessed through a 24-h dietary recall. Participants who reported consuming 2300 mg of sodium or less per day (equivalent to one teaspoon of salt) were classified as having low-to-moderate salt intake. Those who consumed more than 2300 mg/day were considered to have high salt intake (American Diabetes Association, 2020).

#### 2.4.5 Physical activity

Physical activity was measured using self-reported weekly minutes of moderate-to-vigorous exercise. Participants who met the World Health Organization's recommendation of at least 150 min of moderate-intensity or 75 min of vigorous-intensity activity per week were considered physically active; others were labeled inactive (American Diabetes Association, 2020).

#### 2.4.6 Alcohol consumption

Alcohol use was classified based on reported daily intake. Women consuming more than one standard drink per day and men consuming more than two were considered to have high alcohol consumption (American Diabetes Association, 2020). In this study, one standard drink was defined as any beverage containing approximately 14 g of pure alcohol. This is equivalent to a 12-oz can of regular beer (5 % alcohol by volume), a 5-oz glass of wine (12 % alcohol by volume), or a 1.5-oz shot of distilled spirits (40 % alcohol by volume). Locally consumed beverages were also categorized according to their approximate alcohol content.

### 2.5 Data quality assurance

Before the actual data collection, a one-day training was provided to data collectors and supervisors on study objectives, ethical considerations, and data collection procedures. A pre-test involving 5 % of the sample was conducted at a nearby primary hospital to assess the clarity and reliability of the tools. The principal investigator and supervisor conducted daily reviews of completed questionnaires to check for completeness and consistency. Double data entry was performed for a subset of responses to identify entry errors.

### 2.6 Ethical consideration

Ethical clearance was obtained from the Institutional Review Board of Arba Minch University College of Medicine and Health Sciences. A formal permission was also secured from the Arba Minch General Hospital administration. Oral informed consent was obtained from each participant before data collection. Confidentiality and privacy were ensured throughout the study by anonymizing the data and securing all records. Participants were informed of their right to withdraw from the study at any point without consequences. All methods were performed following the Declaration of Helsinki and ethical guidelines for human research.

### 2.7 Statistical analysis

Descriptive statistics such as frequencies, means, and standard deviations were used to summarize participant characteristics. Bivariate logistic regression was used to assess the association between each independent variable and the presence of hypertension. Variables with a *p*-value <0.25 in the bivariate analysis were included in the multivariable logistic regression model to control for potential confounders, including age, sex, educational level, residence, family history of diabetes, glycemic control, body mass index, current smoking, duration of diabetes, physical activity, alcohol consumption, dietary sodium intake, and use of self-monitoring device.

Adjusted odds ratios (AOR) with 95 % confidence intervals (CI) were reported, and a *p*-value <0.05 was considered statistically significant. The Hosmer–Lemeshow goodness-of-fit test assessed model fitness (0.57), and multicollinearity was checked using the variance inflation factor (VIF < 5). Data were entered into EpiData version 4.2 and analyzed using R software version 4.4.1.

## 3 Results

### 3.1 Sociodemographic characteristics of participants

A total of 381 adults with type 2 diabetes mellitus participated in the study, which gives a response rate of 97.7 %. The mean age of participants was 49.06 years (SD ± 15.89), with the majority (68.0 %) aged 40 years and above. More than half were male (52.8 %), and the majority were married (67.7 %).

In terms of education, 33.3 % of participants had completed secondary and above education, and 37.0 % had elementary education. Regarding occupation, the most common categories were government employees (30.2 %), housewives (20.5 %), and merchants (26.0 %). Most participants were urban residents (68.2 %) (Table 1).

**Table 1:** Sociodemographic Characteristics of Patients with Type-2 Diabetes Mellitus at Arba Minch General Hospital, South Ethiopia, 2024 (*n* = 381).

Variable	Categories	Frequency	Proportion
Age	Mean ± SD = 49.06 ± 15.89		
	18–39	122	32.0
	40+	259	68.0
Sex	Male	201	52.8
	Female	180	47.2
Marital status	Married	258	67.7
	Single	98	25.7
	Others*	25	6.6
Educational level	No formal education	113	29.7
	Elementary (Grade 1–8)	141	37.0
	Secondary and above	127	33.3
Occupation	Government employee	115	30.2
	Merchant	99	26.0
	Farmer	42	11.0
	House-wife	78	20.5
	Student	30	7.9
	Others**	17	4.5
Residence	Urban	260	68.2
	Rural	121	31.8

Others* = Widowed, Divorced; Others** = Non-employed, Retired; SD = Standard Deviation.

### 3.2 Clinical characteristics and self-care practice of participants

Most participants (60.6 %) had been living with Type 2 Diabetes Mellitus for less than 10 years, and 37.5 % reported a family history of diabetes. Regarding treatment, 42.8 % used both insulin and oral hypoglycemic agents (OHA), 31.8 % used OHA alone, and 25.5 % used insulin only. Optimal glycemic control was achieved by 32.3 % of participants, 66.9 % were overweight, and 41.7 % had a prior hypertension diagnosis or were on antihypertensive treatment.

In terms of self-care, 33.6 % used a self-monitoring device, 26.5 % were physically active, and 8.1 % were current smokers. Additionally, 60.6 % consumed a recommended amount of dietary salt, and 64.3 % reported alcohol intake within recommended limits (Table 2).

**Table 2:** Clinical Characteristics and Self-Care Practices of Patients with Type-2 Diabetes Mellitus at Arba Minch General Hospital, South Ethiopia, 2024 (n = 381).

Variable	Value	Frequency	Proportion
Family History of Diabetes Mellitus	Yes	143	37.5
	No	238	62.5
Diabetes Mellitus duration, years	Mean ± SD = 8.61 (2.29)		
	< 10 years	231	60.6
	≥ 10 years	150	39.4
Current regimen	Insulin	97	25.5
	Oral Hypoglycemic Agents	121	31.8
	Insulin + OHA	163	42.8
Glycemic control	Optimal	123	32.3
	Suboptimal	258	67.7
Body Mass Index	Normal	126	33.1
	Overweight	255	66.9
Hypertension Self-Reported or on Treatment	Yes	159	41.7
	No	222	58.3
Self-monitoring device	Yes	128	33.6
	No	253	66.4
Current cigarette smoking	Yes	31	8.1
	No	350	91.9
Physical activity	Active	101	26.5
	Not active	280	73.5
Dietary sodium intake	Low-to-moderate	231	60.6
	High	150	39.4
Alcohol consumption	≤2 standard drinks	245	64.3
	High	136	35.7

SD = Standard Deviation; OHA = Oral Hypoglycemic Agents.

“Self-monitoring device” refers to a personal glucometer use for home blood glucose monitoring.

Alcohol consumption: high ≥ 2 standard drinks/day (men) or > 1 (women); one “standard drink” ≈ 14 g pure alcohol.

Dietary sodium intake: low–moderate = ≤2300 mg/day (≈1 tsp), high ≥ 2300 mg/day of dietary salt intake.

### 3.3 Prevalence of hypertension

**Fig. 1:**
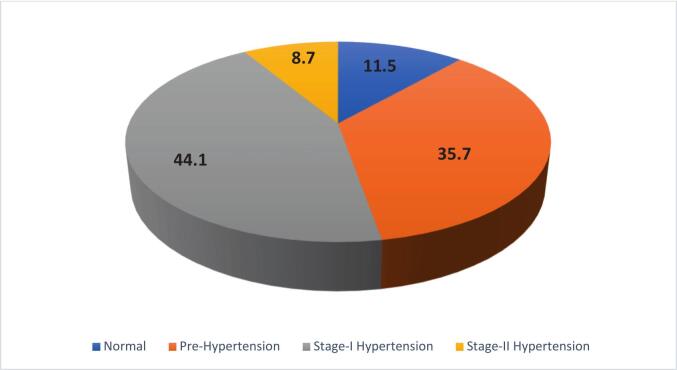
Prevalence of Hypertension Among Patients with Type-2 Diabetes Mellitus at Arba Minch General Hospital, South Ethiopia, 2024.

More than half of the participants (52.8 %; 95 % CI: 47.6, 57.9) were found to have hypertension. Regarding blood pressure stages, 11.5 % of participants had normal blood pressure, 35.7 % were classified as pre-hypertensive, 44.1 % had Stage I hypertension, and 8.7 % had Stage II hypertension (Fig. 1).

### 3.4 Factors associated with hypertension

In the bivariate logistic regression analysis, factors including age, sex, education level, place of residence, family history of diabetes, duration of diabetes, glycemic control, body mass index, current cigarette smoking, alcohol consumption, dietary sodium intake, physical activity, and self-monitoring device use were associated with hypertension. In the multivariable analysis, after adjusting for potential confounders, several variables remained significantly associated with hypertension.

Adults aged 40 years and above were more than three times as likely to have hypertension compared to younger participants (AOR: 3.32; 95 % CI: 1.68,6.56). Those residing in rural areas had significantly lower odds of hypertension compared to urban residents (AOR: 0.41; 95 % CI: 0.19,0.88). A family history of diabetes was independently associated with twice the odds of hypertension (AOR: 2.31; 95 % CI: 1.23,4.45). Diabetes duration of ten or more years was also associated with increased odds (AOR: 3.50; 95 % CI: 1.87,6.65). In addition, physical inactivity (AOR: 2.53; 95 % CI: 1.01,6.55) and not using a self-monitoring device (AOR: 2.11; 95 % CI: 1.13,3.93) were significantly associated with hypertension (Table 3).

**Table 3:** Factors Associated with Hypertension Among Patients with Type-2 Diabetes Mellitus at Arba Minch General Hospital, South Ethiopia, 2024 (n = 381).

Variable	Categories	Hypertension	
		Unadjusted Odds Ratio (95 % CI)	Adjusted Odds Ratio (95 % CI)
Age	18–39	1.00	1.00
	40+	6.47 (3.95, 10.89)	3.32 (1.68, 6.56)
Sex	Male	1.00	1.00
	Female	0.78 (0.52, 1.17)	0.69 (0.34, 1.40)
Educational level	No formal education	1.00	1.00
	Elementary (Grade 1–8)	0.23 (0.10, 0.48)	0.45 (0.17, 1.14)
	Secondary and above	0.09 (0.04, 0.17)	0.42 (0.15, 1.09)
Residence	Urban	1.00	1.00
	Rural	0.50 (0.33, 0.74)	0.41 (0.19, 0.88)
Family History	No	1.00	1.00
	Yes	1.54 (1.02, 2.32)	2.31 (1.23, 4.45)
Diabetes mellitus duration	<10 years	1.00	1.00
	≥10 years	5.99 (3.87, 9.44)	3.50 (1.87, 6.65)
Glycemic control	Optimal	1.00	1.00
	Suboptimal	2.43 (1.60, 3.72)	1.35 (0.68, 2.66)
Body Mass Index	Normal	1.00	1.00
	Overweight	2.50 (1.41, 4.48)	1.11 (0.53, 2.27)
Current smoking	No	1.00	1.00
	Yes	1.71 (0.63, 5.04)	1.16 (0.29, 4.83)
Alcohol consumption	≤2 standard drinks	1.00	1.00
	High	2.64 (1.50, 4.79)	1.93 (0.87, 4.45)
Dietary Sodium Intake	Low-to-moderate	1.00	1.00
	High	1.67 (1.09, 2.57)	1.16 (0.77, 1.74)
Physical Activity	Active	1.00	1.00
	Not Active	3.48 (1.56, 8.04)	2.53 (1.01, 6.55)
Self-monitoring device use	Yes	1.00	1.00
	No	3.54 (2.35, 5.40)	2.11 (1.13, 3.93)

Covariates in the multivariable model: age, sex, educational level, residence, family history of diabetes, glycemic control, body mass index, current smoking, duration of diabetes, physical activity, alcohol consumption, dietary sodium intake, and use of self-monitoring device.

“Self-monitoring device” refers to a personal glucometer use for home blood glucose monitoring.

Alcohol consumption: high ≥ 2 standard drinks/day (men) or > 1 (women); one “standard drink” ≈ 14 g pure alcohol.

Dietary sodium intake: low–moderate = ≤2300 mg/day (≈1 tsp), high ≥ 2300 mg/day of dietary salt intake.

## 4 Discussion

This study assessed the prevalence and associated factors of hypertension among patients with T2DM in South Ethiopia. The findings revealed that more than half of the participants (52.8 %; 95 % CI: 47.6, 57.9) had coexisting hypertension. This is consistent with findings from other parts of Ethiopia (Akalu and Belsti, 2020; Desta et al., 2024; Sheleme et al., 2022), as well as studies from other low-income countries (Mwimo et al., 2023; Boima et al., 2024; Stanikzai et al., 2025). These results indicate the growing burden of hypertension among people with T2DM and the challenges in managing comorbid conditions. The high prevalence of hypertension calls for strengthened integration of chronic disease management into diabetes care. However, our finding is lower than the prevalence rates reported in some studies (Lee et al., 2022; Sun et al., 2021). Differences in healthcare infrastructure, study settings, participant ages, and the use of electronic medical records in those countries may explain this variation.

Among sociodemographic variables, age was significantly associated with hypertension. Participants aged 40 years and above were more than three times more likely to have hypertension compared to younger individuals. This finding supports earlier studies from Ethiopia (Akalu and Belsti, 2020) and other countries (Mwimo et al., 2023; Stanikzai et al., 2025), showing that increasing age is a major non-modifiable risk factor for hypertension in people with T2DM. Age-related physiological changes, such as vascular stiffening, endothelial dysfunction, and reduced nitric oxide availability, can lead to increased peripheral resistance and elevated blood pressure (Ahmed et al., 2024). In addition, older adults are more likely to lead sedentary lifestyles, gain excess weight, and experience insulin resistance (Park et al., 2020). Socioeconomic challenges, including reduced income during retirement, may also limit access to healthy food and healthcare, further increasing the risk (McMaughan et al., 2020).

Urban residence was also significantly associated with a higher prevalence of hypertension compared to rural settings. This supports findings from earlier studies in Ethiopia, suggesting that urbanization contributes to rising rates of non-communicable diseases (Haile et al., 2022). Urban environments typically promote a sedentary lifestyle, increased consumption of processed foods high in salt and unhealthy fats, and greater exposure to stress, all of which contribute to the development of both diabetes and hypertension. A family history of diabetes was also independently associated with nearly twice the odds of hypertension. This finding aligns with other study (Kotiso et al., 2021), and this relationship may reflect shared genetic predispositions and common lifestyle factors such as poor dietary habits and physical inactivity that cluster within families (Ahmed, 2024). These results show the importance of family-based health education and early risk screening among relatives of diabetic patients.

Duration of diabetes was another important factor; those with diabetes for ten years or more had significantly higher odds of hypertension. This association has been documented in previous studies (Akalu and Belsti, 2020; Desta et al., 2024) and is likely due to the cumulative effects of prolonged hyperglycemia, which causes damage to the vascular endothelium, increases oxidative stress, and impairs kidney function (Charlton et al., 2020). Over time, these changes contribute to increased blood pressure and elevate the risk of cardiovascular complications (Charlton et al., 2020). Early detection and continuous management of diabetes are therefore essential to delay the onset of hypertension and its complications.

Finally, behavioral factors such as physical inactivity and the lack of regular self-monitoring device use were significantly associated with hypertension. Despite the recommendations for at least 150 min of moderate aerobic activity (or 75 min of vigorous activity) per week (American Diabetes Association, 2020), only 30 % of our participants met these guidelines. Physical inactivity contributes to weight gain, insulin resistance, and dyslipidemia. Suboptimal self-monitoring device use may also reflect poor engagement in self-care (Yaribeygi et al., 2021). These issues further compound the risk of hypertension and emphasize the importance of patient education and lifestyle modification in reducing comorbid risk.

### 4.1 Limitations of the study

This study has limitations that should be considered when interpreting the findings. First, due to the cross-sectional design, causal relationships between the identified factors and hypertension cannot be established. Second, self-reported data by participants, such as salt intake and alcohol consumption, may be subject to recall or social desirability bias. Third, some potential confounding factors, such as stress levels, were not assessed. Fourth, the study was conducted at a single general hospital, which may limit generalizability to other settings.

Despite the mentioned limitations, this study provides context-specific evidence on hypertension among patients with type 2 diabetes mellitus in a low-resource setting, using standardized measurements and a relatively large sample size. These findings can inform targeted interventions and guide future longitudinal studies.

## 5 Conclusion

This study found that more than half of adults living with T2DM in South Ethiopia had coexisting hypertension, revealing a significant burden of comorbidity. Several factors were associated with hypertension, including older age, urban residence, longer duration of diabetes, family history of diabetes, physical inactivity, and lack of self-monitoring device use. These findings emphasize the need for integrated chronic disease management, with a focus on lifestyle modification, regular screening, and targeted interventions for high-risk groups. Strengthening education on physical activity, dietary habits, and self-care practices, particularly in urban settings and among older adults, could help to reduce long-term complications and improve outcomes in people living with T2DM.

## Authors contribution

BDA conceived the study, designed the study, supervised data collection, analyzed and interpreted the data, and drafted the manuscript. NAN, SMD, HEB, HW, YH, and MDG contributed to data collection, literature review, interpretation, and manuscript revision. All authors read and approved the final manuscript.

## CRediT authorship contribution statement

**Biniyam Demisse Andarge:** Writing – review & editing, Writing – original draft, Visualization, Validation, Supervision, Software, Resources, Project administration, Methodology, Investigation, Formal analysis, Data curation, Conceptualization. **Nini Asfaw Negash:** Writing – review & editing, Investigation, Data curation. **Sayih Mehari Degualem:** Writing – review & editing, Resources, Investigation. **Habtamu Esubalew Bezie:** Writing – review & editing, Resources, Investigation, Data curation. **Habtamu Wondmagegn:** Writing – review & editing, Resources, Investigation, Data curation. **Tsegazeab Ayele Meshesha:** Writing – review & editing, Resources, Investigation, Data curation. **Maycas Dembelu Gembe:** Writing – review & editing, Resources, Investigation, Data curation. **Yohannes Habtegiorgis:** Writing – review & editing, Resources, Investigation, Data curation.

## Funding

This research did not receive any specific grant from funding agencies in the public, commercial, or not-for-profit sectors.

## Declaration of competing interest

The authors declare that they have no known competing financial interests or personal relationships that could have appeared to influence the work reported in this paper.

## Data Availability

Data will be made available on request.
